# The Holocaust, medicine and becoming a physician: the crucial role of education

**DOI:** 10.1186/s13584-019-0327-3

**Published:** 2019-06-27

**Authors:** Shmuel P. Reis, Hedy S. Wald, Paul Weindling

**Affiliations:** 1 Center for Medical Education, Hadassah/Hebrew University Faculty of Medicine, P.O.B 12272, 9112102 Jerusalem, Israel; 2 Department of Family Medicine, Warren Alpert Medical School of Brown University, Providence, RI USA; 3 Wellcome Trust Research Professor in the History of Medicine in the Department of History, Oxford Brookes University, Oxford, UK

**Keywords:** Holocaust and medicine, Medical education, Professional identity formation, Bioethics

## Abstract

Learning about the abandonment of moral principles of healthcare professionals and scientists, their societies and academic institutions, to a murderous ideology yields fundamental concerns and global implications for present and future healthcare professionals’ education and practice. Medicine’s worst-case scenario raises deeply disturbing yet essential questions in the here and now: Could the Holocaust, one of the greatest evils ever perpetrated on humankind, have occurred without the complicity of physicians, their societies, and the scientific profession community? How did healers become killers? Can it happen again?

We reflect here on those queries through the lens of the Second International Scholars Workshop on Medicine during the Holocaust and Beyond held in the Galilee, Israel on May 7–11, 2017 and derive contemporary global lessons for the healthcare professions. Following a brief historical background, implications of the history of medicine in the Holocaust are drawn including 1) awareness that the combination of hierarchy, obedience, and power constitutes a risk factor for abuse of power in medicine and 2) learning and teaching about medicine in the Holocaust and beyond is a powerful platform for supporting professional identity formation. As such, this history ideally can help “equip” learners with a moral compass for navigating the future of medical practice and inherent ethical challenges such as prejudice, assisted reproduction, resource allocation, obtaining valid informed consent, end of life care, and challenges of genomics and technology expansion. Curriculum modules are available and studies on impact on students’ attitudes and behavior are emerging.

The conference culminated with the launch of the Galilee Declaration, composed and signed by an international, inter-professional community of historians, healthcare professions educators, and ethicists. The Declaration included herein (http://english.wgalil.ac.il/category/Declaration) calls for curricula on history of healthcare professions in the Holocaust and its implications to be included in all healthcare professions education.

The Holocaust. A zenith of profound evil afflicted by human beings upon their fellow humans under National Socialism (1940–45) [1, 2]. “Never again” is a resounding promise for a civilized society, and “never again” for the medical profession as well. While many professions swept by Nazi ideology went overboard to please the Führer, physicians and other healthcare professionals were among the most avid [3, 4]. Forty-five percent of German physicians, for example, joined the Nazi party (many of those even before Hitler’s rise to power) compared to 7% of teachers in Germany [4]. Learning about and reflecting upon the abandonment of moral principles of individual healthcare professionals and scientists and more broadly, their societies and academic institutions, to a murderous ideology yields fundamental concerns and global implications for present and future healthcare professionals’ education and practice [5–7]. Medicine’s worst-case scenario [8] raises deeply disturbing yet essential questions in the here and now: Could the Holocaust, one of the greatest evils ever perpetrated on humankind, have occurred without the complicity of physicians, their societies, and the scientific profession community? How did healers become killers? Can it happen again? We reflect here on those queries through the lens of the Second International Scholars Workshop on Medicine during the Holocaust and Beyond held in the Galilee, Israel on May 7–11, 2017 and derive contemporary global lessons for the healthcare professions. This gathering of over 100 historians, physicians, nurses, medical and university educators, medical students, ethicists and more from 17 countries presenting on history, healthcare professions education, and bioethics addressed this dark chapter of history and contemplated its implications for current and future practitioners and researchers.

## Background

Some historical background is presented here as context for this workshop: Five “programs” deliberately launched by the Nazis established the priority of the “health” of the State or “Volk” over the health of individuals and dehumanized those considered “unworthy of life.” first, the Jewish medical presence was eliminated from Nazi Germany (1935–1939). A second program consisted of involuntary sterilization of disabled German citizens (Jews included) (1933–1939), followed by a third, “euthanasia” of (mostly) Germans and Austrians whose lives were deemed medically “unworthy of living,” including the disabled and mentally ill (1939–1945). Fourth, horrific experiments on numerous persons ranging from mixed-race Afro-German children to Jews in Auschwitz were conducted throughout this period (1933–1945) [9] and fifth, medicalized genocide aimed to annihilate European Jewry and other “undesirables” (1941–1945), indeed from 1940 given that Jewish psychiatric patients were prioritized for killing with carbon monoxide gas. These programs were proposed, planned, organized, and implemented (contributed to for the fifth) by healthcare professionals and scientific institutions, implicating almost the entire scientific and medical enterprise of Germany and annexed Austria [6, 7]. The sequence of events was gradual. The first two “programs” enacted prior to World War II rendered an estimated 350,000 persons forcibly sterile and resulted in several thousand Jewish physicians being ousted from their jobs with many committing suicide or eventually perishing in the ghettoes and death camps. In this prelude to what was to follow, German and Austrian physicians and their institutions engaged in reporting, “legally” sanctioning, and executing these first two crimes. “The Holocaust started in the dark basements of German hospitals” [10].

Once the war began on September 1, 1939, Hitler permitted transitioning from sterilization to the T4 (“euthanasia”) program where, once again, German and Austrian disabled individuals were gassed. While this program officially ceased in August, 1941 after public protest with the Catholic Bishop Galen taking a lead, medical teams continued to perform “wild euthanasia” on a decentralized basis similar to the “special care children’s units” until late May, 1945. During 1941, the infrastructure, personnel and expertise of the T4 program were transferred east to serve the emerging “Final Solution” (plan to annihilate European Jewry officially launched in January 1942), by building the first three extermination camps on German-occupied Polish soil: Sobibor, Belzec, and Treblinka, as well as testing Zyklon gas for killing sick Poles and Russian prisoners in September 1941 in Auschwitz before large scale killing of Jews from 1942. It was also in Auschwitz where sick prisoners were routinely killed with lethal injections or sent to the gas chambers. Intermediary steps toward achieving the Holocaust included massive population transfers to ghettoes and labor camps fueled by medical advice on such actions “preventing epidemics” [11] and genocide by bullets in territories seized from the Soviet Union during the 1941–44 German invasion. In parallel, experimentation on ca 18,000 prisoners and patients, with medical and scientific establishments competing for generous research grants offered to support war related and racial research [12]. The pre-existing German Medical Ethics Guidelines of 1931 did not prevent such abuse and violation of human rights [13]. In the death camps, physicians conducted a disinformation charade intended to mislead with white coats and ambulances on the train ramps, followed by the infamous selection of immediately “diagnosing” the few who may be fit for work and sending the vast majority of deportees to gas chambers and crematoria. The image of the “angel of death” Josef Mengele MD, PhD looms large as the icon of medical presence selecting on the ramp of Auschwitz a select minority for forced labor and most for immediate killing by poison gas, as well as twins and persons with genetic growth defects for coerced experiments and extraction of soft tissue, body fluids and bones [4]. Mengele did not act alone. Research implicates numerous German and Austrian medical physicians and others in the scientific community as participating in this unprecedented breach of ethics and humanity [14].

Additional issues raised during the conference included the involvement of pharma in exploiting available slave labor and participating in unethical experiments, examples of the international medical community and its journals giving voice to, supporting, and publishing policies, ideology and “scientific” results of the Nazi medical and scientific communities, failures of compliance in many countries with ethical codes formulated in the aftermath of the Holocaust, and finally denial with transparency obstruction of the medical atrocities and their perpetrators in involved countries. Essential lessons for health professionals in all stages of the professional life cycle derive from studying history highlighting what may occur when ideology fuels evil distortion of medicine and science. The conference confronted the history of the often-central role of Nazi healthcare professionals in atrocities including exclusion of persons categorized as racially “sub humans” from the realm of ethical consideration, human beings labeled as unworthy of living [15], starvation and other forms of “legally” administered torture, and barbaric experiments conducted in the ruthless pursuit for “medical knowledge.”

### Implications of the history of medicine in the Holocaust for medical ethics and education

Potential for abuse of power is inherent to medicine. Healthcare can involve the application of aggressive, potentially painful and distressing modalities, i.e. delivering bad news, surgery, resuscitation, distressing and often painful diagnostic and therapeutic procedures. Physicians hold the power to announce and certify death, assist in reproductive technologies, admit against a patient’s will, and supply a host of certificates and reports which profoundly affect patients’ lives and welfare. Medicine is a hierarchical profession, with senior clinicians issuing orders to be carried out by junior ones, and where physicians often direct or command allied health personnel [16]. While these features of medicine are applied with the noble goal of healing and administering best practices within humanistic care, the combination of elements of hierarchy, obedience, and power constitutes a risk factor for abuse of power [2, 16]. What prevents potentialities for abuse of power in medicine from becoming reality?

Such existential deliberations were integrated at the conference within medical education imperatives, i.e. considering how the history of medicine under the Third Reich informs and shapes contemporary clinical ethics, human subject experimentation checks and balances, and required ethics committees, ethics consults, and institutional review boards (IRBs). An ethics code or instruction cannot suffice – “medical ethics” in Nazi-era medical school curricula existed, yet included “unequal worth of human beings, authoritative role of the physician, and priority of public health over individual patient care” [17, 18] Nazi physicians, idealistic in a perverse way, earnestly believed what they were doing was right [19]. While no physician was present in the Wannsee meeting that formally confirmed the plan for genocide of the Jewish people, [20] programs 1–4 as described above could not have been realized without individual physicians and the medical and scientific communities playing a decisive role in the planning and implementation of such programs. As for program #5, most horrific of all, the question of whether the Holocaust could have occurred without the contributions of the medical and scientific professions is haunting. Nearly 100 conference presentations included topics such as the medical/scientific community’s involvement in medical atrocities and bioethics after the Holocaust as well as medical activity (including ethical dilemmas) in ghettos and camps, genocide conditions, treatment of survivors and their offspring, resilience and post-trauma [21].

Learning and teaching about medicine in the Holocaust and beyond is a powerful platform for supporting professional identity formation. As such, this history ideally can help “equip” learners with a moral compass for navigating the future of medical practice and inherent ethical challenges such as prejudice, assisted reproduction, resource allocation, obtaining valid informed consent, end of life care, and challenges of genomics and technology expansion [7]. In addition to its relevance within overt racism and prejudice, medicine in the Holocaust pedagogy is germane to increasing awareness of potential for implicit bias to shape healing relationships, albeit in subtle ways, and efforts to identify, reduce, and ideally eliminate this characteristic within healthcare professions practice [22, 23].

Fostering reflection on the history of the profession has the potential to broaden learners’ perspectives, raise awareness, and promote empathy, compassion, and tolerance of the “other” [6, 24]. Medical student-authored publications have highlighted the contemporary relevance of Holocaust and Medicine curriculum for professional development [25, 26] as “hard truths are faced,” i.e. “What exactly did Nazi physicians do; how are their actions a part of Western medicine’s legacy [25]; and what can we do as a profession to prevent a repeat?”

Within this, there is increasing attention to the valuable contribution of the history of medicine within the medical humanities to medical education [27]. The inclusion of examples of altruism, deep humanity, and devotion to medicine as a calling while risking one’s life and the life of one’s family during the Holocaust can serve as inspiration [5–7]. Jewish prisoner healthcare professionals as well as “righteous of the nations’ ones” cared for patients, taught junior learners, conducted research, and manifested resistance to evil even under brutal conditions and in mortal danger [28–30]. The Warsaw Ghetto heroic Jewish healthcare system, its clandestine medical school, and the Hunger Study [31, 32] as well as Ludwik Fleck’s typhus vaccine sabotage, and the White Rose student resistance group based in the Munich medical school are some specific exemplars [28–30].

Holocaust and Medicine curriculum modules are integrated into health professions curricula [6], with further examples included at http://www.medicineaftertheholocaust.org/category/curricula-syllabi/. Studies of curriculum impact on students’ attitudes and behavior are emerging [33] including within a 3 year credit-earning elective curriculum for interprofessional students (including study excursions to Holocaust sites; students may participate in any/all of the years) at the Witten-Herdecke University Faculty of Health-Integrated Curriculum for Anthroposophic Medicine (ICURAM) in collaboration with the Ita Wegman Institute (https://ibam.uni-wh.de/en/, http://www.wegmaninstitut.ch/).

The Scholars Workshop on Medicine during the Holocaust and Beyond culminated with the launch of the Galilee Declaration, composed and signed by an international, inter-professional community of historians, healthcare professions educators and ethicists (additional signatures invited.) The Declaration (included below) and accessed at: http://english.wgalil.ac.il/category/Declaration) calls for curriculum on history of healthcare professions in the Holocaust and its implications to be included in all healthcare professions education. The document emulates the 2000 Stockholm Declaration [34] that transformed general Holocaust education worldwide, with the intention of similar impact.

## Conclusions: what global lessons can we draw from this critical reflection?

It is about the major ethical questions of our time: the soul of care, equity in healthcare, resource allocation, pharma ethical conduct, non-exploitation of the vulnerable, poor and marginalized, rights of the disabled, beginning and end of life issues…and more. It is about current and future health professionals being cognizant of the inherent danger of abuse of power in medicine and the need for “immunizing” ourselves for prevention. Challenges abound within the practice of healing, given reality of violence [35], stress, fatigue, burnout-inducing systems factors [36] and potential dilemmas of dual commitment (eg. physicians in uniform) [37]. It is about critical aspects of professionalism, professional identity formation, and prevention of abuse of power. These include desired professional competencies of conscience, empathy, compassion, reflection, and resilience (emotional and moral) in the individual health professional in conjunction with additional technical competencies. It is also about checks and balances, remembering and commemorating, learning and teaching within the shared humanity of individuals, institutions, societies, and nations.

May we carry Primo Levi’s words as an everlasting reminder: “It can happen, and it can happen everywhere” [38].

## Data Availability

NA

## Abbreviation

T4: The Nazi “euthanasia” program (1939–41) in which Germans & Austrians whose lives were considered “unworthy of living” were gassed. T4 stands for the address of the office overseeing this program
